# Significant benefits of adding neoadjuvant chemotherapy before concurrent chemoradiotherapy for locoregionally advanced nasopharyngeal carcinoma: a meta-analysis of randomized controlled trials

**DOI:** 10.18632/oncotarget.10237

**Published:** 2016-06-23

**Authors:** Mengmeng Wang, Huimin Tian, Gang Li, Tingwen Ge, Yudi Liu, Jiuwei Cui, Fujun Han

**Affiliations:** ^1^ Cancer Center, The First Hospital of Jilin University, Changchun, China; ^2^ Department of Thyroid and Breast Surgery, No.458 Hospital of People's Liberation Army, Guangzhou, China

**Keywords:** adjuvant chemotherapy, concurrent chemoradiotherapy, meta-analysis, nasopharyngeal carcinoma, neoadjuvant chemotherapy

## Abstract

**Purpose:**

We did a meta-analysis to compare the efficacy and safety of neoadjuvant chemotherapy (NACT) followed by concurrent chemoradiotherapy (CCRT) versus CCRT with or without adjuvant chemotherapy (AC) for patients with locoregionally advanced nasopharyngeal carcinoma based on randomized controlled trials.

**Methods:**

We searched PubMed, Embase, Web of Science, ClinicalTrials.gov, Chinese National Knowledge Infrastructure, and meeting proceedings of major relevant conferences to identify published and unpublished randomized controlled trials. Progression-free survival (PFS) was the primary endpoint.

**Results:**

This meta-analysis included 9 randomized clinical trials with 2215 patients. NACT followed by CCRT significantly improved PFS (HR=0.68, 95% CI 0.56 – 0.81, *P* < 0.001) compared versus CCRT with or without AC, and no heterogeneity was observed (I^2^ = 0.0%, *P* = 0.975). NACT was associated with a significant improvement in overall survival (HR = 0.64, 95% CI 0.49 – 0.84, *P* = 0.001; I^2^ = 0.0%, *P* = 0.467) and distant failure-free survival (HR = 0.72, 95% CI 0.53 – 0.97, *P* = 0.031; I^2^ = 0.0%, *P* = 0.744). No significant benefit was shown by NACT for locoregional control. NACT with CCRT increased risks of grade 3 – 4 anemia, thrombocytopenia, leukopenia, and fatigue, compared versus CCRT with or without AC.

**Conclusions:**

Our meta-analysis confirmed that the addition of NACT to CCRT significantly improved PFS and OS *versus* CCRT with or without AC for locoregionally advanced nasopharyngeal carcinoma. These results may alter the standard of care - CCRT with or without AC, for locoregionally advanced nasopharyngeal carcinoma.

## INTRODUCTION

Nasopharyngeal carcinoma displays a marked geographic distribution, with the highest incidence in Southern China. Radiotherapy is the cornerstone of treatment for nasopharyngeal carcinoma. Radiotherapy alone is recommended for patients who present with T1, N0, M0 disease [1]. In more advanced stages (T1, N1–3 and T2–T4, any N lesions), the standard of care is concurrent chemoradiotherapy (CCRT) with or without adjuvant chemotherapy (AC) [1–3].

The National Comprehensive Cancer Network has widespread disagreement on the role of neoadjuvant chemotherapy (NACT) in the treatment of locoregionally advanced nasopharyngeal carcinoma [1]. A recent meta-analysis demonstrated that NACT followed by CCRT or radiotherapy could improve both overall survival (OS) and progression-free survival (PFS) for patients with locoregionally advanced nasopharyngeal carcinoma, compared with CCRT or radiotherapy [4]. However, the benefit could possibly relate to the superiority of NACT followed by radiotherapy over radiotherapy alone. Consequently, it remains unclear whether or not the addition of NACT before CCRT is better than the standard approach of CCRT with or without AC. Four meta-analyses directly [5–6] or indirectly [7–8] showed that NACT plus CCRT did not improved OS compared versus CCRT or CCRT with AC in patients with locoregionally advanced nasopharyngeal carcinoma. However, the largest pair-wise comparison included only 4 trials with a total of 798 patients [6], so these meta-analyses were underpowered by a small sample size [5–8]. In addition, the primary endpoint of all these meta-analyses was OS [5–8]. The longest median follow-up in the included trials was 4.6 years [9], whereas the 5-year survival rate was about 70% for the advanced disease [1, 10]. As a result, the duration of follow-up might not have been long enough to determine the effect of NACT on OS. It is hence unclear whether NACT followed by CCRT is better than the standard of care - CCRT with or without AC, for patients with locoregionally advanced nasopharyngeal carcinoma.

The gold standard endpoint for clinical trials of nasopharyngeal carcinoma was OS [11]. However, with augmented applications of secondary and tertiary treatments for nasopharyngeal carcinoma, PFS can be considered as a better primary endpoint than OS [12]. Moreover, PFS appears concordant with OS in trials of combined chemotherapy and radiotherapy for nasopharyngeal carcinoma [11]. In addition to the trials included in the previous meta-analyses, additional randomized studies on the benefit of NACT plus CCRT compared versus CCRT with or without AC in nasopharyngeal carcinoma have been available [13–17]. To determine the effect of NACT before CCRT for locoregionally advanced nasopharyngeal carcinoma, we performed this meta-analysis with PFS as the primary endpoint.

## RESULTS

### Trials

**Figure 1:**
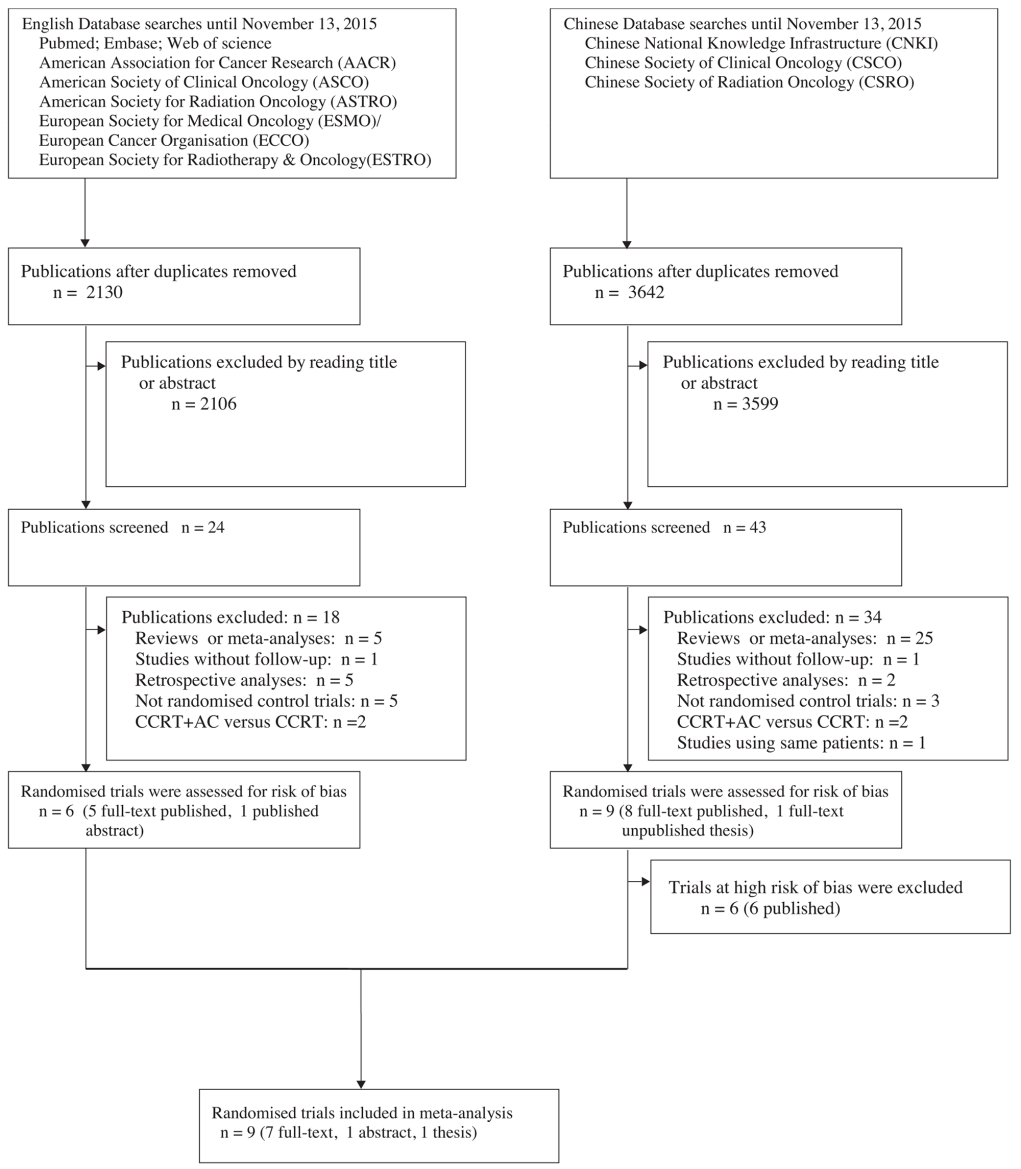
Flow chart showing inclusion and exclusion of trials

The initial search using four English databases (ClinicalTrials.gov, PubMed, EMBASE, and Web of Science) yielded 2130 publications dated until November 13, 2015. Of these, 2106 publications were irrelevant by title or abstract reading. After full text reading, 6 trials were identified to meet the inclusion criteria [9, 13–14, 18–20]. We also found 9 randomized trials in the Chinese literature [15–17, 21–26]. (Figure 1) As a result, a total of 15 randomized trials were identified. Two trials [13, 16] included 3 and 6 intervention arms, respectively. For each trial, we combined the interventions into one treatment arm to compare to one control arm, according to the recommendation proposed by the Cochrane Handbook for Systematic Reviews of Interventions [27].

### Assessing risk of bias in included trials

The assessments of risk of bias for each individual trial were described in Supplementary Figure S1. 6 trials were graded as high risk of bias, and were excluded from this meta-analysis [21–26]. The characteristics of these trials were listed in Supplementary Table S1. The other 9 trials were graded as unclear risk of bias, and were eligible for a meta-analysis [9, 13–20]. The characteristics of these trials were listed in Table 1.

**Table 1:** Description of trials included in the meta-analysis

First author, Year, (reference)	Race(Region)	Clinical stage (TNM classification)	Histology(WHO classification)	Radiotherapy	Concurrent chemoradiotherapy	Induction chemotherapy	Adjuvant chemotherapy	Patients randomized (treatment/control)	Median follow-up(month)
Fountzilas et al.,2012, [9]	Greek(Greece)	AJCC/UICC 6th edition IIB-IVB	1-3	(79 patients) 3D-CRT and (62 patients) 2D-CRT: 2.0Gy/F×5F/wk; primary site 66-70 Gy; positive nodes 66-70 Gy; pharyngeal extension and residual nodes 50 Gy.	Cisplatin 40 mg/m^2^ d1, q1wk×8	Cisplatin 75 mg/m^2^ d1; Epirubicin 75 mg/m^2^ d1; Paclitaxel 175 mg/m^2^ d1; q3wks×3		72/69	55 (0.5 – 76.2)
Tan et al. 2015, [18]	Singaporean(Singapore)	UICC/AJCC 5th edition T3-4NxM0 or TxN2-3M0	2,3	(168 patients) IMRT: GTVnx: 69.96Gy/2.12Gy/33F; GTVnd: 69.96Gy/2.12Gy/33F; CTV1: 60Gy/1.82Gy/33F. (4 patients) 2D-CRT: primary site 70Gy/2Gy/35F; positive nodes 70Gy/2Gy/35F; pharyngeal extension and residual nodes 60Gy/2Gy/30F.	Cisplatin 40 mg/m^2^ d1, q1wk×8	Paclitaxel 70mg/m^2^ d1, d8; Carboplatin AUC=2.5 d1, d8; Gemcitabine 1000mg/m^2^ d1, d8; q3wks×3		86/86	40.8(13.2 - 100.8)
Ma et al. 2014, [14]	Chinese(Mainland China)	UICC/AJCC 7th edition III-IVB (except T3-4N0)	2,3	IMRT (radical radiotherapy)	Cisplatin 100 mg/m^2^ d1, q3wks×3	Docetaxel 60mg/m^2^ d1; Cisplatin 60mg/m^2^ d1; Fluorouracil 600 mg/m^2^ d1-5; q3wks×3		241/239	18.6 (0.8 - 34)
Hui et al. 2009, [19]	Chinese(Hong Kong)	UICC/AJCC 5th edition III-IVB	NR	(17 patients) IMRT and (48 patients) 2D-CRT: 2 Gy/F×5F/wk; total 66 Gy; residual boost of 7.5 Gy.	Cisplatin 40 mg/m^2^ d1, q1wk×8	Docetaxel 75 mg/m^2^ d1; Cisplatin 75 mg/m^2^ d1; q3wk×2		34/31	51.6
Huang et al. 2012, [17]	Chinese Mainland (China)	AJCC/UICC 6th edition III-IVB	2,3	2D-CRT: 2.0Gy/F×5F/wk; primary site 65-78 Gy; positive nodes 60-70 Gy; pharyngeal extension and residual nodes 50-54 Gy.	Caboplatin AUC = 6 d7, d28, d49	Caboplatin AUC=6 d1; Fluorouracil 750mg/m^2^, d1-5; q3wks×2		100/100	46.8
Gao et al. 2013, [15]	Chinese(Mainland China)	1992 Fuzhou stage T3-4N2-3M0	2,3	2D-CRT: 2.0Gy/F×5F/wk; primary site 70-74 Gy; positive nodes 66-70 Gy; pharyngeal extension and residual nodes 50 Gy.	Cisplatin 40 mg/m^2^ d1, q1wk×7	Cisplatin 30mg/m^2^ d1-3; Fluorouracil 450mg/m^2^ d1-3; q3wks×2		57/55	42 (> 24)
Sun, 2009, [16]^a^	Chinese(Mainland China)	1992 Fuzhou stage III-IVA	2,3	(156 patients) IMRT: 5F/wk; GTVnx: 68Gy/30F; GTVnd: 60 - 66Gy/30F; CTVl: 60Gy/30F; CTV2: 54Gy/30F. (57 patients) 2D-CRT: 2.0Gy/F×5F/wk; primary site 70 Gy; positive nodes 66-70 Gy; pharyngeal extension and residual nodes 50 Gy.	Cisplatin 80mg/m^2^ d1, q3wks×2	Group 1 (76 patients): Cisplatin 80mg/m^2^ d1; Fluorouracil 1.5g/m^2^ d1-2; q3wks×2 Group 2 (66 patients) : Fluorouracil 1.5g/m^2^ d1-2; Carboplatin AUC = 6; q3wks×2		142/71	26.3 (2.5 - 44.7)
Lee et al, 2015, [13]^b^	Chinese(Hong Kong and Mainland China)	AJCC/UICC 6th edition III-IVB	2,3	IMRT^c^ 2.0Gy/F×5F/wk or 2.0Gy/F×6F/wk; gross tumor target 70 Gy; positive nodes <70 Gy; pharyngeal extension and residual nodes 50 Gy.	Cisplatin 100 mg/m2 d1, q3wks×2/3	Cisplatin 100 mg/m2 d1, Fluorouracil 1g/ m2 d1-5, q3wks×2/3; or Cisplatin 100 mg/m2 d1, capecitabine 2g/ m2 d1-14, q3wks×2/3	Cisplatin 80 mg/m2 d1, Fluorouracil 1g/ m2 d1-4, q4wks×3	538/264	39.6(1.2-85.2)
Ruste et al, 2011, [20]	Philippinese(Philippines)	III-IVB	2,3	2D-CRT: 2.0Gy/F×5F/wk; primary site 70Gy, N0 disease 50Gy, nodes<2cm 66 Gy, nodes greater than 2cm 70Gy.	Cisplatin 25 mg/m2 d1-4, q3wks×3	Cisplatin 20 mg/m2 d1-4, q4wks×3; 5-Fluorouracil 1000 mg/m2 d1-4; q4wks×3	Cisplatin 20 mg/m2 d1-4; 5-Fluorouracil 1000 mg/m2 d1-4; q4wks×3	14/16	19(8-30)

a Patients were allocated to 6 arms: arm 1A (conventional fractionation radiotherapy, adjuvant chemotherapy using Cisplatin plus 5-Fluorouracil ), arm 2A (conventional fractionation radiotherapy, neoadjuvant chemotherapy using Cisplatin plus 5-Fluorouracil), arm 3A (conventional fractionation radiotherapy, neoadjuvant chemotherapy using Cisplatin plus Capecitabine), arm 1B (accelerated fractionation radiotherapy, adjuvant chemotherapy using Cisplatin plus 5-Fluorouracil), arm 2B (accelerated fractionation radiotherapy, neoadjuvant chemotherapy using Cisplatin plus 5-Fluorouracil), arm 3B (accelerated fractionation radiotherapy, neoadjuvant chemotherapy using Cisplatin plus Capecitabine). In this meta-analysis, we combined arm 2A, arm 3A, arm 2B, and arm 3B together as a treatment group, and combined arm 1A and arm 1B together as a control group.

b Patients were divided into three study groups, comparing concurrent chemoradiotherapy (control group) with two different NACT plus CCRT treatments (treatment group). For the purpose of the present meta-analysis, the control group was directly used, and the two treatment groups were pooled together. 2D-CRT, Two-dimensional conformal radiation therapy; 3D-CRT, Three-dimensional conformal radiation therapy; AJCC, American Joint Committee on Cancer; AUC, area under the curve; CTV, clinical target volume; F, fraction; GTV, Gross tumor volume; IMRT, intensity-modulated radiotherapy; TNM, Tumour Nodes Metastasis; UICC, International Union Against Cancer; WHO, World Health Organization; d, day; q1wk, every 1 week; q3wk, every 3 weeks; wk, week.

c 95% patients were treated with IMRT.

### Progression-free survival

The median follow-up ranged from 18.6 to 55 months. One trial was from non-endemic region (Greece) [9], and the others were from endemic regions [13–20]. Seven trials (1383 patients) investigated NACT plus CCRT versus CCRT alone [9, 14–19]. Two trials (832 patients) investigated NACT plus CCRT versus CCRT plus AC [13, 20].

**Figure 2:**
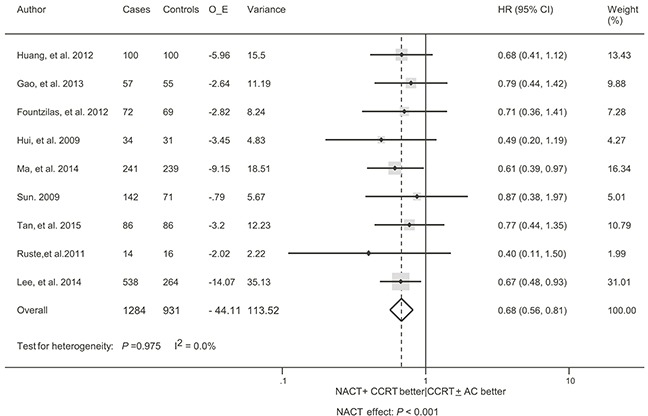
Forest plot for the hazard ratio of progression-free survival with neoadjuvant chemotherapy followed by concurrent chemoradiotherapy versus concurrent chemoradiotherapy with or without adjuvant chemotherapy for locoregionally advanced nasopharyngeal carcinoma AC, adjuvant chemotherapy; CCRT, concurrent chemoradiotherapy; CI, confidence interval; HR, hazard ratio; NACT, neoadjuvant chemotherapy; O-E, observed minus expected events.

The meta-analysis of PFS was based on 9 trials with 2215 patients [9, 13–20]. The addition of NACT improved PFS (Hazard ratio [HR] = 0.68, 95% confidence interval [CI] 0.56 – 0.81, *P* < 0.001; Figure 2). No heterogeneity was observed among trials (*P* = 0.975, I^2^ = 0.0%), confirming the appropriateness of pooling the data. The leave-1-out sensitivity analysis showed that no single trial exerted a significant influence on this result, indicating that the result was reliable. Subgroup analyses were also conducted in order to check whether features of the included trials affected the result of this meta-analysis (Table 2). The association between NACT and an improved PFS was maintained regardless of duration of follow-up, sample size, CCRT timing, radiotherapy technique, NACT regimen, method of data extraction, and with or without AC. The HRs ranged from 0.64 to 0.72, and there was no evidence of heterogeneity (I^2^ = 0.0%) in all subgroups, suggesting a small variability in the effect of NACT across different inclusion criteria for trials. No significant differences in treatment effect were found across subgroups (*P* -value for interaction > 0.05; Table 2).

**Table 2:** Subgroup analyses for the treatment effect on progression-free survival of neoadjuvant chemotherapy followed by concurrent chemoradiotherapy versus concurrent chemoradiotherapy with or without adjuvant chemotherapy for locoregionally advanced nasopharyngeal carcinoma

Factors	Subgroups	Availability		Effect		Heterogeneity		Interaction	
		Trials (*N*)	Patients (*N*)	HR (95% CI)	*P* value	I^2^	*P* value	*P* value
Duration of follow-up								
	>36 months	6	1492	0.69(0.56-0.85)	0.001	0.0	0.966	0.748
	<36 months	3	723	0.64(0.43-0.93)	0.020	0.0	0.585	
Sample size								
	<150 patients	4	348	0.66(0.45-0.97)	0.033	0.0	0.944	0.915
	>150 patients	5	1867	0.68(0.55-0.84)	0.000	0.0	0.706	
CCRT timing ^a^								
	q1wk	4	490	0.72(0.52-0.97)	0.045	0.0	0.833	0.669
	q3wk	5	1725	0.66(0.53-0.83)	0.000	0.0	0.889	
Method of data extraction^b^								
	Directly reported	5	1660	0.66(0.53-0.82)	0.000	0.0	0.927	0.791
	Indirect method	4	555	0.72(0.51-0.98)	0.048	0.0	0.771	
Radiotherapy technique^c^								
	Conventional radiotherapy	4	407	0.66(0.47-0.92)	0.015	0.0	0.713	0.872
	IMRT/3DCRT	5	1808	0.69(0.55-0.85)	0.001	0.0	0.943	
IC regimen								
	Two drugs	6	1422	0.68(0.54-0.85)	0.001	0.0	0.882	0.952
	Three drugs	3	793	0.68(0.50-0.93)	0.015	0.0	0.810	
	Taxol-included	4	858	0.65(0.49-0.88)	0.005	0.0	0.876	0.832
	Non-taxol-included	5	1357	0.69(0.55-0.88)	0.002	0.0	0.832	
AC								
	With	7	1383	0.65(0.47-0.90)	0.008	0.0	0.959	0.812
	Without	2	832	0.70 (0.55-0.87)	0.005	0.0	0.453	
Data source								
	Published	7	1522	0.68(0.55-0.84)	<0.001	0.0	0.954	0.904
	Unpublished	2	693	0.66(0.45-0.99)	0.043	0.0	0.459	

a Including one trial in which chemotherapy was administered every 4 weeks [20].

b HR and its 95% CI were directly reported or indirectly calculated according to the method by Parmar et al. [46] in a trial.

c Radiotherapy technique was classified as conventional radiotherapy or IMRT/3D-CRT based on the radiotherapy applied for at least 70% of the study population. 3D-CRT, Three-dimensional conformal radiation therapy; AC, adjuvant chemotherapy; CCRT, concurrent chemoradiotherapy; CI, confidence interval; DFFS, distant failure-free survival; HR, hazard ratio; NACT, neoadjuvant chemotherapy; IMRT, intensity-modulated radiotherapy; q1wk, every 1 week; q3wk, every 3 weeks.

### Overall survival

**Figure 3:**
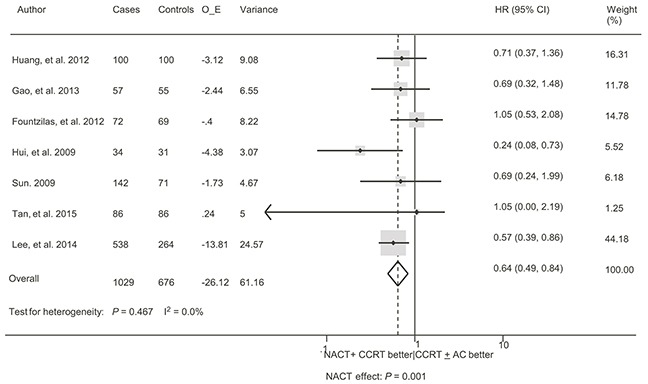
Forest plot for the hazard ratio of overall survival with neoadjuvant chemotherapy followed by concurrent chemoradiotherapy versus concurrent chemoradiotherapy with or without adjuvant chemotherapy for locoregionally advanced nasopharyngeal carcinoma See Figure 2 for abbreviations.

Two trials (510 patients), with *a median follow-up period of less than* 2 years [14, 20], were excluded because they did not fulfill our eligibility criteria. The meta-analysis included 7 trials with 1705 patients [9, 13, 15–19]. Overall, NACT before CCRT provided a significant benefit in OS compared versus CCRT with or without AC (HR = 0.64, 95% CI 0.49 – 0.84, *P* = 0.001; Figure 3). There was no evidence of heterogeneity between trials (I^2^ = 0.0%, *P* = 0.467). The sensitivity analysis demonstrated that no single trial exerted a significant influence on the overall result. Table 3 shows the results of the subgroup analyses. We identified possible evidence of heterogeneity in subgroup analyses (n≥2) when the trials were divided based on NACT regimen (two drugs versus three drugs). Notably, any subgroup with more than 800 patients showed a statistically significant association, whereas no association was shown in only subgroups with fewer than 600 patients. Therefore, the negative results in these subgroups might be attributable to a lack of statistical power (a small sample size) to detect the effect size. No significant interaction was observed between subgroups (*P* -value for interaction > 0.05; Table 3). The strongest interaction was between NACT regimen and OS: two-drug NACT was more efficient than three-drug NACT (ratio of HR = 0.56, 95% CI 0.27 −1.15, *P* = 0.119).

**Table 3:** Subgroup analyses for the treatment effect on overall survival of neoadjuvant chemotherapy followed by concurrent chemoradiotherapy versus concurrent chemoradiotherapy with or without adjuvant chemotherapy for locoregionally advanced nasopharyngeal carcinoma

Factors	Subgroups	Availability		Effect		Heterogeneity		Interaction	
		Trials (*N*)	Patients (*N*)	HR (95% CI)	*P* value	I^2^	*P* value	*P* value
Duration of follow-up								
	<48 months	4	1327	0.62(0.47-0.84)	0.002	0.0	0.927	0.960
	>48 months	3	378	0.60(0.19-1.87)	0.380	59.6	0.084	
Radiotherapy technique^a^								
	Conventional radiotherapy	3	377	0.59(0.38-0.92)	0.021	32.7	0.226	0.285
	IMRT/3DCRT	4	1328	0.67(0.49-0.93)	0.015	0.0	0.492	
Sample size								
	<150 patients	3	318	0.62(0.29-1.32)	0.216	59.0	0.087	0.999
	>150 patients	4	1387	0.62(0.45-0.85)	0.003	0.0	0.912	
CCRT timing^b^								
	q1wk	4	490	0.65(0.34-1.25)	0.198	39.5	0.175	0.872
	q3wk	3	1215	0.61(0.45-0.84)	0.003	0.0	0.826	
Method of data extraction^c^								
	Directly reported	4	1180	0.61(0.44-0.85)	0.003	0.0	0.999	0.755
	Indirect method	3	525	0.70(0.45-1.09)	0.112	44.1	0.147	
IC regimen								
	Two drugs	5	1392	0.59(0.44-0.78)	0.000	0.0	0.543	0.119
	Three drugs	2	313	1.05(0.54-2.04)	0.885	0.0	1.000	
	Taxol-included	3	378	0.60(0.20-1.87)	0.380	59.6	0.084	0.962
	Non-taxol-included	4	1327	0.62(0.47-0.84)	0.002	0.0	0.927	
AC								
	With	1	802	0.64(0.49-0.83)	0.005	-	-	0.798
	Without	6	903	0.70(0.50-0.98)	0.040	0.0	0.420	
Data source								
	Published	6	1492	0.64(0.48-0.87)	0.004	10.2	0.351	0.913
	Unpublished	1	213	0.69(0.25-1.93)	0.883	-	-	

a Radiotherapy technique was classified as conventional radiotherapy or IMRT/3D-CRT based on the radiotherapy applied for at least 70% of the study population.

b HR and its 95% CI were directly reported or indirectly calculated according to the method by Parmar et al. [46] in a trial.

c Including one trial in which chemotherapy was administered every 4 weeks[20].

See Table 2 for abbreviations.

### Distant failure-free survival and locoregional failure

**Figure 4:**
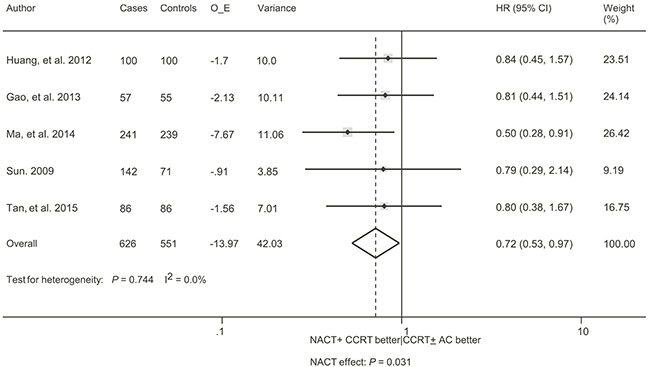
Forest plot for the hazard ratio of distant failure-free survival with neoadjuvant chemotherapy followed by concurrent chemoradiotherapy versus concurrent chemoradiotherapy with or without adjuvant chemotherapy for locoregionally advanced nasopharyngeal carcinoma See Figure 2 for abbreviations.

**Figure 5:**
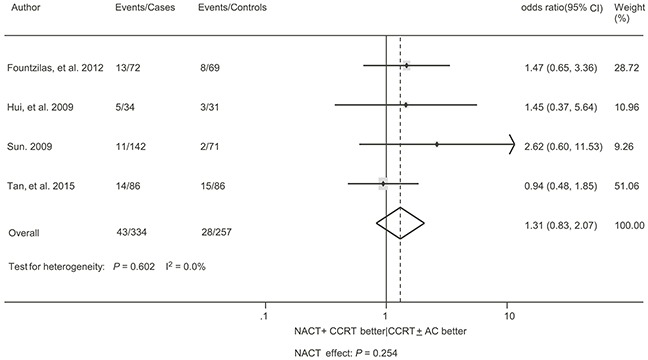
Forest plot of the odds ratio of locoregional failure with neoadjuvant chemotherapy followed by concurrent chemoradiotherapy versus concurrent chemoradiotherapy with or without adjuvant chemotherapy for locoregionally advanced nasopharyngeal carcinoma See Figure 2 for abbreviations.

As for distant failure-free survival (DFFS), the meta-analysis included 5 trials with 1177 patients [14–18]. A significant benefit in favor of the addition of NACT was found without evidence of heterogeneity (HR = 0.72, 95% CI, 0.53 – 0.97, *P* = 0.031; I^2^ = 0.0%, *P* = 0.744; Figure 4). The sensitivity analysis demonstrated the trial by Ma et al. [14] exerting a significant influence on the overall result. The HR was non-significant (HR = 0.82, 95% CI, 0.57 – 1.16, *P* = 0.255; I^2^ = 0.0%, *P* = 0.999) when this trial was excluded. Data regarding the absolute number of locoregional failure were available in 4 trials with 591 patients [9, 16, 18–19]. There was no benefit in favor of the addition of NACT, without evidence of heterogeneity (odds ratio [OR] = 1.31, 95% CI, 0.83 – 2.07, *P* = 0.254; I^2^ = 0.0%, *P* = 0.602; Figure 5). The sensitivity analysis showed that no single trial exerted a significant influence on the overall result.

### Treatment-related adverse events

Table 4 described the acute and late grade 3 – 4 *adverse events* that were observed in these trials. The most common grade 3 – 4 acute adverse events were leukopaenia (22.5%), mucositis (17.9%), and esophagitis (15.4%). 20 acute adverse events, which were reported by more than 2 trials, were meta-analyzed. 7 acute adverse events, including anemia, dermatitis, mucositis, and nausea/vomiting, thrombocytopenia, leucopenia, and nephrotoxicity were reported by more than 5 trials with a sample size of more than 860. The addition of NACT before CCRT was associated with a significantly increased risk of anemia (OR = 1.87, 95% CI 1.04-3.34, *P* = 0.036; I^2^ = 7.8%, *P* = 0.370), thrombocytopenia (OR = 3.67, 95% CI 1.85–7.23, *P* < 0.001; I^2^ = 14.6%, *P* = 0.319), leucopenia (OR = 2.13, 95% CI 1.00–4.57, *P* = 0.050; I^2^ = 70.8%, *P* = 0.004), and fatigue (OR = 2.47, 95% CI 1.03–5.94, *P* = 0.043; I^2^ = 49.4%, *P* = 0.139), compared versus CCRT with or without AC. The incidences of other acute adverse events were comparable between the two arms. There was some evidence of heterogeneity between trials in the acute adverse event analyses. 6 late adverse events, which were mainly related to radiotherapy, were reported by at least 2 trials. The incidences of the late adverse events were not statistically different between the two arms. Because most of these meta-analyses included a small number of samples, a lack of statistical power should be considered.

**Table 4:** Severe adverse events of neoadjuvant chemotherapy followed by concurrent chemoradiotherapy versus concurrent chemoradiotherapy with or without adjuvant chemotherapy for locoregionally advanced nasopharyngeal carcinoma

Adverse events	Availability			Effect		Heterogeneity	
	Trials (*N*)	Cases (events/total)	Control (events/total)	OR (95% CI)	*P* value	I^2^	*P* value
Acute adverse events							
Anemia	8	38/948	14/602	1.87 (1.04, 3.34)	0.036	7.8	0.370
Dermatitis	6	28/986	31/646	0.83 (0.50, 1.38)	0.474	44.1	0.111
Mucositis	7	163/1020	140/672	1.02 (0.76, 1.37)	0.914	69.1	0.004
Nausea/vomiting	8	52/1034	57/688	0.71 (0.36, 1.41)	0.325	53.6	0.035
Thrombocytopenia	7	39/1020	7/672	3.67 (1.85, 7.23)	0.000	14.6	0.319
Leukopenia	6	127/462	68/398	2.13 (1.00, 4.57)	0.050	70.8	0.004
Nephrotoxicity	6	11/806	10/531	0.81 (0.35, 1.86)	0.613	0.0	0.936
Fatigue	3	17/183	6/182	2.47 (1.03, 5.94)	0.043	49.4	0.139
Hepatotoxicity	3	5/243	3/241	1.44 (0.43, 4.89)	0.581	0.0	0.876
Xerostomia	4	11/305	13/243	0.87 (0.39, 1.92)	0.730	29.7	0.234
Esophagitis	2	19/149	28/156	0.69 (0.36, 1.30)	0.251	0.0	0.698
Hoarseness	2	6/149	6/156	1.05 (0.36, 3.05)	0.934	0.0	0.535
Neurotoxicity	3	0/163	1/172	0.65 (0.08, 4.99)	0.676	0.0	0.884
Anorexia	2	4/77	4/86	1.08(0.28, 4.20)	0.911	0.0	0.450
Electrolyte disturbance	2	12/572	12/290	0.67(0.28, 1.57)	0.353	0.0	0.367
Diarrhea	2	2/77	0/86	3.37(0.34, 33.27)	0.298	0.0	0.533
Dysphagia	3	21/615	17/350	1.00(0.52, 1.92)	0.991	9.8	0.330
Infection	2	3/601	5/334	0.43(0.12, 1.52)	0.190	0.0	0.320
Weigh loss	3	21/625	23/350	0.92(0.48, 1.77)	0.806	0.0	0.744
Otitis	2	3/152	2/159	1.36 (0.30, 6.20)	0.695	0.0	0.920
Late adverse events							
Xerostomia	2	15/120	17/112	0.70 (0.33, 1.52)	0.373	14.3	0.280
Mucositis	2	3/123	2/114	1.08 (0.04, 30.56)	0.962	58.8	0.119
Otitis	2	10/572	9/290	0.60(0.25, 1.42)	0.248	37.0	0.208
Subcutaneous fibrosis	2	9/120	3/112	2.28 (0.66, 7.82)	0.194	0.0	0.545
Dermatitis	2	4/120	5/112	0.66 (0.19, 2.35)	0.522	0.0	0.827
Esophagitis	2	1/121	0/112	1.68 (0.15, 19.36)	0.676	0.0	0.748

AC, adjuvant chemotherapy; CCRT, concurrent chemoradiotherapy; CI, confidence interval; NACT, neoadjuvant chemotherapy; OR, odds ratio.

### Publication bias

Asymmetry in the funnel plot was not observed in the meta-analysis of the effect of NACT on PFS (Supplementary Figure S2). No publication bias was suggested by Begg's rank correlation test (*P* = 0.999) and Egger's linear regression test (*P* = 0.581). Tests of publication bias were not conducted for the other meta-analyses because too few studies were available to make a valid statistical test.

## DISCUSSION

Compared with previous meta-analyses [5–8], this study included more trials (Table 5), and therefore was able to employ rigorous methodology to estimate the effect of NACT on locoregionally advanced nasopharyngeal carcinoma. The results of this meta-analysis suggested that the advantages of NACT followed by CCRT over CCRT with or without AC might be real in locoregionally advanced nasopharyngeal carcinoma. We confirmed an improvement in PFS and OS by the addition of NACT before CCRT. We also showed benefits in DFFS favoring the addition of NACT, whereas no benefit in locoregional control was shown.

**Table 5:** Pair-wise comparisons of neoadjuvant chemotherapy followed by concurrent chemoradiotherapy versus concurrent chemoradiotherapy with or without adjuvant chemotherapy for locoregionally advanced nasopharyngeal carcinoma in the previous and current meta-analyses

	Overall survival			Progression-free survival			Localregional failure-free survival			Distant failure-free survival		
Meta-analysis	Trials(*N*; size)	HR(95%CI)	Heterogeneity(I^2^, %)	Trials(*N*, size)	HR(95%CI)	Heterogeneity(I^2^, %)	Trials(*N*, size)	HR(95%CI)	Heterogeneity(I^2^, %)	Trials(*N*, size)	HR(95%CI)	Heterogeneity(I^2^, %)
Yan et al, 2015,[8]^a^	3; 378	0.88(0.57–1.36)	NI	NI	NI	NI	NI	NI	NI	NI	NI	NI
Song et al, 2015, [6]	3; 318	0.52(0.21–1.29)	61.9	4; 798	**0.66(0.49–0.90)**	0.0	NI	NI	NI	2; 592	**0.60(0.39–0.98)**	0.0
Liang et al, 2013, [5]^b^	3; 371	0.99(0.72-1.36)	0.0	2; 95	**0.37(0.20-0.69)**	0.0	3; 347	1.08(0.84-1.38)	34.0	2; 287	0.98(0.75-1.27)	23.0
Chen et al, 2015,[7]^a^	2; 206	0.70(0.39-1.26)	79.0	NI	NI	NI	2; 206	1.65(0.95-2.86)	0.0	2; 206	**0.51(0.28-0.95)**	0.0
The present study^c^	7; 1705	**0.64(0.49 – 0.84)**	0.0	9; 2215	**0.68(0.56 – 0.81)**	0.0	4; 591	1.31(0.83 – 2.07)	0.0	5; 1177	**0.72(0.53 – 0.97)**	0.0

a Shown was the pair-wise comparison included in the network meta-analysis.

b The selected effect size for overall survival, progression-free survival, locoregional failure-free survival, and distant failure-free survival was relative risk with 95% CIs.

c The selected effect size for locoregional failure-free survival was odds ratio with 95% CIs.

CI, confidence interval; HR, hazard ratio; NI, not investigated.

Consistent with previous studies [5–6], this meta-analysis showed that the addition of NACT before CCRT significantly improved PFS. The improvement in PFS was consistent across all trials [9, 13–20], despite a marked variability in terms of NACT regimen, duration of follow-up, radiotherapy technique, method of data extraction, and staging system for nasopharyngeal carcinoma in the included trials. The variability was equivalent to adding “noise” to the analysis. It was likely that an association might be weakened or masked by noise [28]. In spite of the variability, the association between a significant improvement in PFS and NACT was also seen consistently across different patient subgroups, and there was no evidence of heterogeneity in all the meta-analyses. The extensive consistency provided optimal evidence of the credibility of an association [29] between NACT and an improved PFS. In addition, the credibility could be further strengthened by the clinical variability. Therefore, our meta-analysis provided strong evidence that NACT followed by CCRT improved PFS when compared to CCRT with or without AC in patients with locoregionally advanced nasopharyngeal carcinoma.

The results of this meta-analysis of OS were related with two important clinical issues. First, NACT followed by CCRT had shown a marked improvement in OS compared versus CCRT with or without AC. The present meta-analysis had the largest sample size up-to-date and the sufficient statistical power to detect the treatment effect, whereas previous meta-analyses failed to do so [5–8]. This was in line with the results of our subgroup analyses: no significant benefit in OS in all small patient subgroups (n < 800), but a consistently significant benefit in OS in all large patient subgroups (n > 800). We identified potential evidence of heterogeneity when trials were stratified based on NACT regimens (two-drug NACT or three-drug NACT). Two-drug NACT regimen was shown to be better in OS compared with three-drug NACT. Although the difference was not statistically significant, the power of this test was low [30], and a genuine difference could not be ruled out. Because this result was opposite to that found for head and neck cancer [31–33], but was in agreement with that found for lung cancer [34–35]. This proposed the second important clinical issue. This issue might be solved by two ongoing randomized, controlled Phase III trials (NCT01536223: docetaxel plus cisplatin and 5-fluorouracil versus cisplatin and 5-fluorouracil; NCT02016417: docetaxel plus cisplatin and 5-Fluorouracil versus gemcitabine and cisplatin).

Our meta-analysis showed an improvement in DFFS, but not in the incidence of locoregional failure, with the incorporation of NACT. These results were in line with previous reports [6, 36]. Regarding DFFS, the leave-1-out sensitivity analysis showed that the positive finding relied heavily on the trial conducted by Ma et al. [14]. The meta-analysis of the remaining trials was unable to show a significantly improved DFFS favoring NACT, but each individual trial consistently showed such a trend. These could be an indication of a small but a real benefit in DFFS. Furthermore, Ma et al.'s trial was a large and well-designed multicenter randomized controlled study (ClinicalTrials.gov, NCT01245959) [14], the inclusion of this trial was necessary. Taken together, these suggested a genuine benefit in DFFS favoring NACT for locoregionally advanced nasopharyngeal carcinoma.

A concern for the addition of NACT is an increase of severe acute *adverse events*, which in turn compromises the delivery of subsequent CCRT. The present meta-analysis showed that the addition of NACT was mainly associated with an increased risk of severe *hematologic adverse events*, including leukopenia and thrombocytopenia. These acute adverse events were uncomplicated and manageable with growth factor support.

This meta-analysis had several limitations: (1) Due to the lack of individual patient data, we were not able to check each trial to apply consistent conditions for inclusion and to standardize analysis techniques in this meta-analysis. However, literature based meta-anlayses are often consistent with those based on individual patient data [37], and should not be viewed as “inferior.” [38]. (2) Although the tests for publication bias did not identify major publication bias, these might not have enough power unless the number of included studies was more than 10 [39]. Our subgroup analyses indicated that small studies did not show significantly larger effects than large studies. In addition, according to the Venice criteria *that were developed to assess* cumulative evidence of genetic associations, a small effect size (such as, OR < 1.15 or > 0.87) might be vulnerable to biases [29, 40–41]. Our meta-analyses showed much larger effect sizes (a HR of 0.68 for PFS and a HR of 0.64 for OS). These results suggested that publication bias might not be a significant threat to our meta-analyses. (3) The duration of follow-up was short in most trials included in this meta-analysis. Although there is evidence that PFS is predictive of OS for a number of cancer types (nasopharyngeal carcinoma [11] and bladder cancer [42]), a re-analysis of the data after longer follow-up will enable us to better assess the treatment effect on OS. (4) Due to the incomplete data inclusion, the results of the meta-analyses on DFFS and locoregional failure should be viewed as exploratory only. The use of locoregional failure rate to compute OR instead of the use of HR might result in bias [43]. (5) Regarding most adverse events, especially late adverse events, the large amount of missing data did not allow for any meaningful analysis.

In conclusion, this meta-analysis confirmed that NACT followed by CCRT provided a significantly improved PFS compared versus CCRT with or without AC in patients with locoregionally advanced nasopharyngeal carcinoma. We also showed that the addition of NACT was associated with a significant benefit in OS. Although longer follow-up is needed for a better assessment of OS, it is reasonable to recommend the addition of NACT to CCRT for patients with locoregionally advanced nasopharyngeal carcinoma.

## MATERIALS AND METHODS

### Selection criteria

This meta-analysis was done based on a pre-specified protocol. To be eligible, trials needed to compare NACT plus CCRT versus CCRT with or without AC in previously untreated patients with histologically proven nasopharyngeal carcinoma without distant metastases. NACT, CCRT, and AC were defined as chemotherapy administered before, during, and after radiotherapy, respectively. Only randomized controlled trials were eligible for inclusion. Trials needed to provide data on PFS. Trials without sufficient data for quantitative estimates were listed in the summary overview but were not subjected to a meta-analysis. According to the Cochrane Handbook for Systematic Reviews of Interventions, including trials at high risk of bias may lower the quality of evidence in a meta-analysis [27]. Therefore, risk of bias for each identified trial was assessed, and trials at high risk of bias were excluded. Because patients with locoregionally advanced nasopharyngeal carcinoma who received CCRT had a two-year survival rate of more than 90% [22, 44], a minimum of 2 years follow-up was required for the meta-analysis of OS. Published and unpublished trials were eligible. There were no language restrictions. Case reports, editorials, meta-analyses, and review articles were excluded.

### Literature search strategy

Following the Cochrane Handbook for Systematic Reviews of Interventions [27] and the Preferred Reporting Items for Systematic Reviews and Meta-Analyses guidelines [45], we conducted a comprehensive search of the literature before November 13, 2015. The following databases were used: PubMed, Web of Science, ClinicalTrials.gov, EMBASE, and Chinese National Knowledge Infrastructure databases. A search of the Proceedings of the Annual Meetings of the American Society of Clinical Oncology, American Association for Cancer Research, American Society for Radiation Oncology, European Society for Medical Oncology/European Cancer Organisation, European Society for Radiotherapy & Oncology, Chinese Society of Clinical Oncology, and Chinese Society of Radiation Oncology was conducted to identify relevant studies published in abstract form. In addition, we manually screened citation lists of the retrieved articles to ensure a wider search. The following search terms were used: (chemotherapy) AND (nasopharyngeal OR nasopharynx) AND (cancer OR carcinoma OR neoplasm OR tumor OR malignancy OR malignant) AND radiotherapy.

### Data extraction

Two investigators extracted the following data independently from each individual trial: first author, publication year, region where research was conducted, ethnicity, number of patients, histologic type (WHO criteria), TNM stage, follow-up duration, treatment protocol, compliance with treatment, response to treatment, exclusion (yes/no) from trial analysis and reason for exclusion, and failure pattern.

HR and its 95%CI were directly used if these values were reported in a trial. Otherwise, two investigators calculated the data independently according to the method by Parmar et al. [46] Whenever there were missing data, study authors were contacted via e-mail. Disagreement was resolved by discussion between authors.

### Risk of bias assessments

Risk of bias was independently assessed by two authors, based on the guidelines outlined in the Cochrane Handbook for Systematic Reviews of Interventions Version 5.1.0 [27]. Disagreements were resolved by consensus with a third author. Briefly, each trial was assessed for the following domains: random sequence generation, allocation concealment, blinding of participants and personnel, blinding of outcome assessment, incomplete outcome data, selective reporting, and other sources of bias. Each domain was defined as having a low, high, or unclear risk for bias. It was impossible to blind study participants and personnel to whether or not NACT had been undertaken, and how this influenced the outcome of the clinical trial was not known. Therefore, the domain for all trials was categorized as unclear risk of bias. A trial was considered to have a low risk of bias if all criteria were “low”, an unclear risk of bias if any criteria were “unclear”, and a high risk of bias if any criteria were “high”.

### Subgroup analysis and sensitivity analysis

If there was more than 6 trials included in a meta-analysis, subgroup analyses were conducted. The subgroup analysis was aimed at exploring whether the treatment effect of NACT was stable or dependent on features of the included trials. For this purpose, we predefined the following subgroups based on: duration of follow-up, radiotherapy technique (conventional radiotherapy versus 3D-CRT or IMRT), NACT regimen (two drugs versus three drugs; taxol-included and non-taxol-included), CCRT timing (q1wk versus q3wk), sample size, method of data extraction (directly reported versus indirectly extracted), source of data (published versus unpublished), and AC (with versus without).

For sensitivity analysis, we excluded 1 trial at a time and analyzed the remaining trials to explore whether the results were influenced by a particular trial.

### Outcomes

The main study end point was PFS. Secondary end points were OS, DFFS, the incidence of locoregional failure, and the incidence of treatment-related *adverse events*. All time-to-event variables were calculated from date of randomization. PFS was defined as time to date of progression (locoregional failure or distant failure) or death (whichever occurred first). OS was defined as time to date of death from any cause. DFFS was defined as time to date of distant failure. If a distant failure and a locoregional failure occurred in a patient at the same time, the patient was considered as having an event for a distant failure only.

### Statistical analysis

An intent-to-treat analysis was applied for this meta-analysis [47]. HRs with 95% CIs were used to express results regarding PFS, OS, and DFFS. HRs were calculated using a fixed-effect model. A HR less than 1 suggested an improved survival for NACT plus CCRT treatment compared versus CCRT with or without AC treatment. For locoregional failure and treatment-related adverse events, most reports provided only the absolute number of events, and there was no information available to calculate the HR. Therefore, odds ratios (ORs) was used as the summary statistic for the comparison between groups. The heterogeneity between trials was investigated by using the Cochrane Q test and the I^2^ statistic. A random-effects model (DerSimonian and Laird method [48]) was used in case of obvious heterogeneity (the *P* value of the Cochrane Q test was <0.10 or the I^2^ value was >50%); otherwise, a fixed-effects model (Mantel and Haenszel [49]) was applied. The test of interaction proposed by Altman et al. [30] was used to compare differences in treatment effect across subgroups. Publication bias was evaluated by visualizing the symmetry of the funnel plot and by Begg's rank correlation test and Egger's linear regression test. [50–51] We used Stata software (StataCorp), version 12. Statistical significance was defined as a *P* value of <0.05 (two-sided).

## SUPPLEMENTARY FIGURES AND TABLES




