# Supplementary material for: Significant benefits of adding neoadjuvant chemotherapy before concurrent chemoradiotherapy for locoregionally advanced nasopharyngeal carcinoma: a meta-analysis of randomized controlled trials
Source: Oncotarget. 2016 Jun 23;7(30):48375–90. doi: 10.18632/oncotarget.10237 (PMC5217024; doi:10.18632/oncotarget.10237)
Supplement: Supplementary file 1 [file oncotarget-07-48375-s001.pdf]

Significant benefits of adding neoadjuvant chemotherapy before concurrent chemoradiotherapy for locoregionally advanced nasopharyngeal carcinoma: a meta-analysis of randomized controlled trials

SUPPLEMENTARY FIGURES AND TABLES

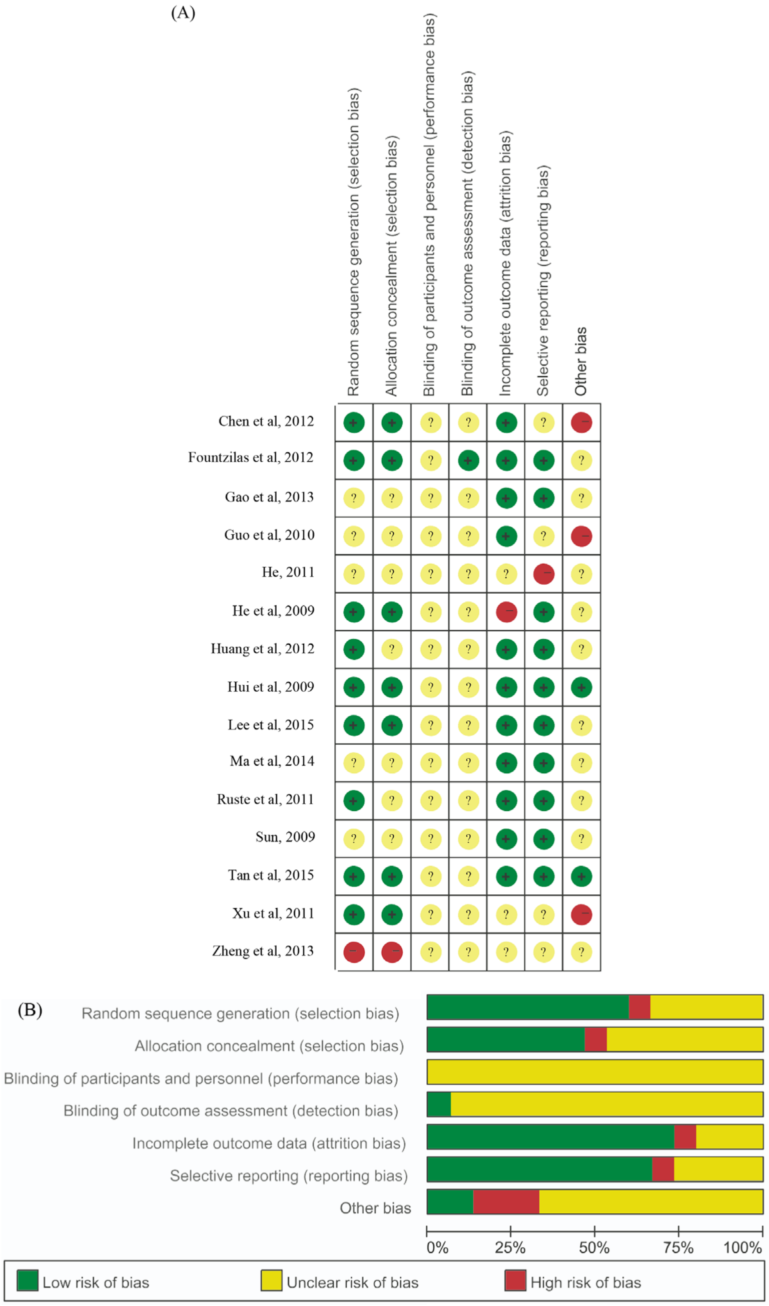

Supplementary Figure S1: Risk of bias summary by study **A.** and by domain **B.** 6 trials were graded as high risk of bias, and were excluded from this meta–analysis [21-26].

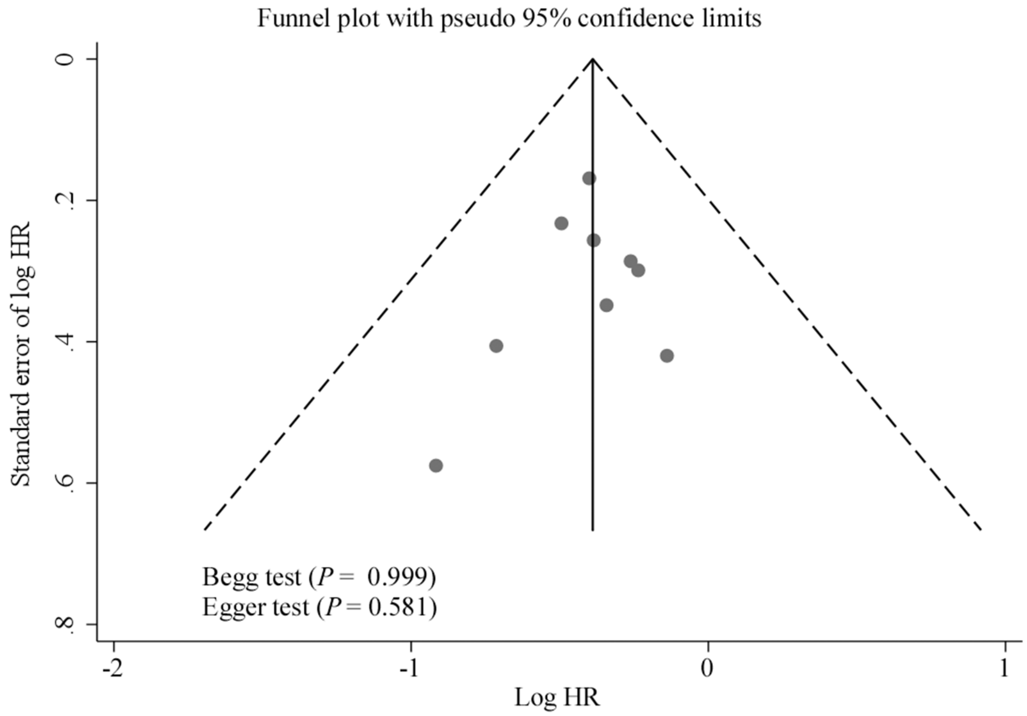

Supplementary Figure S2: Funnel plot for publication bias in meta-analysis of the effect of neoadjuvant chemotherapy on progression-free survival for locoregionally advanced nasopharyngeal carcinoma.

Supplementary Table S1: Description of trials excluded from the meta-analysis.

See Supplementary File 1

Supplementary Table S2: The influence of the radiotherapy fractionation (conventional and accelerated) in Lee's trial [13][a] on the meta-analysis of progression-free survival and overall survival

Factor	PFS				OS			
	HR (95%CI)	*P* value	I^2	*P* value	HR (95%CI)	*P* value	I^2	*P* value
Excluding Lee's trial [13][a]	0.60 (0.32-0.87)	<0.001	24.6	*0.249*	0.70 (0.50-0.98)	0.040	0.0	0.420
Including Lee's trial [13][a]								
Unadjusted								
Conventional group[b]	0.65 (0.51-0.83)	<0.001	0.0	0.919	0.69 (0.52-0.93)	0.013	0.0	0.545
Accelerated group[c]	0.70 (0.55-0.90)	0.005	0.0	0.717	0.73 (0.54-0.98)	0.035	0.0	0.533
Overall	0.70 (0.57-0.86)	0.001	0.0	0.815	0.71 (0.55-0.92)	0.010	0.0	0.640
Adjusted								
Conventional group[b]	0.63 (0.50-0.81)	<0.001	0.0	0.924	0.66 (0.50-0.90)	0.007	0.0	0.505
Accelerated group[c]	0.69 (0.54-0.88)	0.003	0.0	0.792	0.71 (0.53-0.97)	0.029	0.0	0.545
Overall	0.68 (0.56-0.81)	<0.001	0.0	0.975	0.64 (0.49-0.84)	0.001	4.7	0.467

[a] Patients in Lee's trial[13] were allocated to 6 arms: arm 1A (conventional fractionation radiotherapy, adjuvant chemotherapy[AC]), arm 2A (conventional fractionation radiotherapy, neoadjuvant chemotherapy[NACT]), arm 3A (conventional fractionation radiotherapy, NACT), arm 1B (accelerated fractionation radiotherapy, AC), arm 2B (accelerated fractionation radiotherapy, NACT), arm 3B (accelerated fractionation radiotherapy, NACT).
[b] Conventional group: Arms 2-3A vs 1A
[c] Accelerated group: Arms 2-3B vs 1B
CI, confidence interval; HR, hazard ratio; overall survival; PFS, progression-free survival.
