# Supplementary material for: Significant benefits of adding neoadjuvant chemotherapy before concurrent chemoradiotherapy for locoregionally advanced nasopharyngeal carcinoma: a meta-analysis of randomized controlled trials
Source: Oncotarget. 2016 Jun 23;7(30):48375–90. doi: 10.18632/oncotarget.10237 (PMC5217024; doi:10.18632/oncotarget.10237)
Supplement: Supplementary file 2 [file oncotarget-07-48375-s002.doc]

Supplementary Table S1. Description of trials excluded from the meta-analysis.

| First author, Year, (reference) | Race(Region) | Stage (TNM classification) | Histology(WHO classification) | Radiotherapy | Concurrent chemoradiotherapy | Induction chemotherapy | Adjuvant chemotherapy | Patients randomized (treatment/control) | Median follow-up(month) |
| --- | --- | --- | --- | --- | --- | --- | --- | --- | --- |
| He, 2011,[24] | Chinese(Mainland China) | AJCC 2002  III-IVb | 2,3 | 2D-CRT: Median total dose: 70Gy/6.5 weeks | Cisplatin 90mg/m² d1, q3wks×3 | Cisplatin 25mg/m² d1-3; Fluorouracil 800mg/m² d1-4; Docetaxel 75 mg /m^2^ d1; q3wks×3 | - | 50/50 | 24 |
| He et al, 2009, [25] | Chinese(Mainland China) | 1992 Fuzhou stage  III and IVa | 2,3 | 2D-CRT: 2.0Gy/F×5F/wk; primary site 68-72Gy/34-36F; positive nodes 64-66Gy/32-33F; pharyngeal extension and residual nodes 50Gy/25F. | Cisplatin 40mg/m^2^ d1, q1wk×6 | Cisplatin 80mg/m² d1; Fluorouracil 800mg/m² d1-5; q3wks×2 | - | 44/39 | 26.7 (6 – 48) |
| Guo et al, 2010, [22] | Chinese(Mainland China) | 1992 Fuzhou stage  T3-4N2-3M0 | 2,3 | 2D-CRT: 2.0Gy/F×5F/wk; primary site 68-72 Gy; positive nodes 68-70 Gy; pharyngeal extension and residual nodes > 50 Gy. | Cisplatin 40mg/m² d1-3, q3wks×2 | Cisplatin 40mg/m² d1-3; Docetaxel 75mg/m² d1; q3wks×2 | - | 51/63 | 16 (7.7 - 33.3) |
| Zheng et al, 2013, [26] | Chinese(Mainland China) | Chinese 2008 Staging System  IIb-IVa | 2,3 | IMRT: 5F/wk; GTVnx, GTVnd: 66-72Gy/30F; CTV1: 60Gy/30F; CTV2: 54Gy/30F. | Cisplatin 40mg/m² d1, q1wk×6 | Cisplatin 20 mg/m^2^ dl-5, Fluorouracil 500 mg/m2, dl-5; q3wks×1 | - | 37/36 | 26 (15 - 41) |
| Xu et al, 2011, [21] | Chinese(Mainland China) | Chinese 2008 Staging System  IVa(T4 N0-3 M0, T1-4 N3M0) | 2,3 | IMRT: 5F/wk; GTVnx: 70.4-76.4Gy/32-35F; GTVnd:68Gy/32F; CTV1: 60-62Gy/30-32F; CTV2:54-57Gy/30-32F. | Cisplatin 40mg/m^2^ d1, q1w×6 | Cisplatin 75mg/m² d1; Fluorouracil 500mg/m² d1-5; Docetaxel 75 mg /m^2^ d1; q3wks×2 | - | 25/20 | 13.1 (12 - 21) |
| Chen, et al. 2012, [23] | Chinese(Mainland China) | Chinese 2008 Staging System  Ⅲ - Ⅳa | 2,3 | IMRT: 5F/wk; GTVnx: 66.0-70.4Gy/30-32F; GTVnd: 66.0-70.4Gy/30-32F; CTV1: 60.0-64.0Gy/30-32F; CTV2:50.0-54.0Gy/30-32F. | Cisplatin 40 mg/m^2^ d1, q1wk×6 | Cisplatin 75mg/m² d1; Fluorouracil 500mg/m² d1-5; Docetaxel 75 mg /m^2^ d1; q3wks×2 | - | 30/30 | 24 - 36 |

2D-CRT, Two-dimensional conformal radiation therapy; 3D-CRT, Three-dimensional conformal radiation therapy; AJCC, American Joint Committee on Cancer; AUC, area under the curve; CTV, clinical target volume; F, fraction; GTV, Gross tumor volume; IMRT, intensity-modulated radiotherapy; TNM, Tumour Nodes Metastasis; UICC, International Union Against Cancer; WHO, World Health Organization; d, day; q1wk, every 1 week; q3wk, every 3 weeks; wk, week.
